# Global and Comparative Proteome Signatures in the Lens Capsule, Trabecular Meshwork, and Iris of Patients With Pseudoexfoliation Glaucoma

**DOI:** 10.3389/fmolb.2022.877250

**Published:** 2022-04-20

**Authors:** Prity Sahay, Munmun Chakraborty, Aparna Rao

**Affiliations:** ^1^ Hyderabad Eye Research Foundation (HERF), L.V. Prasad Eye Institute, Bhubaneswar, India; ^2^ KIIT School of Biotechnology, Bhubaneswar, India

**Keywords:** pseudoexfoliation, pseudoexfoliation glaucoma, lens capsule, lysyl oxidase, trabecular meshwork, iris, mass spectroscopy, extracellular matrix

## Abstract

Pseudoexfoliation (PXF) is characterized by the accumulation of the exfoliative material in the eye and high rates of blindness if left untreated. Pseudoexfoliation glaucoma (PXG) is generally diagnosed too late due to its asymptomatic nature, necessitating the development of new effective screening tools for the early diagnosis of the disease. Thus, the increasing prevalence of this disease due to an aging population has demanded the identification of suitable biomarkers for the early detection of the disease or detection of the onset of glaucoma in the eyes with PXF. We applied a proteomics strategy based on a high-throughput screening method for the determination of proteins involving PXF and PXG pathogenesis. The lens capsule (LC), iris, and trabecular meshwork (TM) samples with PXF and PXG were taken by surgical trabeculectomy, and control samples were taken from the donor corneal buttons obtained from the institutional eye bank to characterize the proteome profile. Peptides from the LC were analyzed using liquid chromatography with tandem mass spectrometry (LC-MS/MS). The protein of interest and cytokine/chemokine profiles were verified using immunohistochemistry and the bio-plex kit assay, respectively. There were a total of 1433 proteins identified in the human LC, of which 27 proteins were overexpressed and eight proteins were underexpressed in PXG compared with PXF. Overexpressed proteins such as fibromodulin, decorin, lysyl oxidase homolog 1, collagen alpha-1(I) chain, collagen alpha-3(VI) chain, and biglycan were the major components of the extracellular matrix (ECM) proteins involved in cell-matrix interactions or ECM proteoglycans and the assembly and cross-linking of collagen fibrils. The ECM composition and homeostasis are altered in glaucoma. Thus, quantitative proteomics is a method to discover molecular markers in the eye. Monitoring these events can help evaluate disease progression in future studies.

## Introduction

Glaucoma is an age-related progressive neurodegenerative disease and is a cause of chronic irreversible blindness across the globe (Quigley and Green, 1979; Resnikoff et al., 2004; Rao and Padhy, 2014; Chitranshi et al., 2018). Increased intraocular pressure (IOP) is a key modifiable risk factor for glaucoma. Increased IOP, via reducing tissue perfusion and mechanical axoplasmic stasis, causes progressive damage to the optic nerve (Anderson and Hendrickson, 1974; Minckler et al., 1977; Quigley and Green, 1979; Sossi and Anderson, 1983; Ritch and Schredardt, 2001; Izzotti et al., 2003; Fuchshofer and Tamm, 2012; Zhao et al., 2016; Chitranshi et al., 2018). Several retinal and optic neural neuron alterations mark the onset of progressive changes that include interrupted axoplasmic flow, neurofilament aggregation, and extracellular matrix (ECM) remodeling. These changes, coupled and triggered by excitotoxicity, depletion of neurotrophic factors, oxidative damage, and mitochondrial dysfunction, cause irreversible functional damage in the retinal ganglion cells and trabecular meshwork (Anderson and Hendrickson, 1974; Minckler et al., 1977; Quigley and Green, 1979; Sossi and Anderson, 1983). Pseudoexfoliation (PXF) syndrome, a common recognizable cause of secondary open-angle glaucoma, is characterized by the presence of white fibrillar deposits throughout the anterior segment of the eye, specifically the anterior lens capsule, the pupillary margin of the iris, the cornea, and the iridocorneal angle (Ritch and Schredardt, 2001). It is estimated that 15% of the cases of PXF have a probability of developing pseudoexfoliation glaucoma (PXG), although this differs in different ethnic populations (Fuchshofer and Tamm, 2012). The primary, degenerative/ischemic, or mechanical/metabolic damage can result in alterations in the ECM and trabecular network (TM), leading to irreversible retinal ganglion cell degeneration (Izzotti et al., 2003; Tezel, 2006; Babizhayev, 2012; Zhao et al., 2016). Importantly, several studies have shown a link between oxidative stress biomarkers and glaucoma progression (Bhattacharya et al., 2013; Lopez-Riquelme et al., 2015; Sahay et al., 2017; Sahay et al., 2021).

The use of “omics” for disease pathogenesis or staging is not new and has been studied in several groups (Wang-Su et al., 2003; Bai et al., 2020; Rao et al., 2021; Semba et al., 2021). Multiomics studies will enable significant progress in the understanding of disease at multiple levels in the coming years, which are as follows: i) identification of biomarkers to be used in the diagnosis or prognosis of the disease, ii) advancement in the knowledge of possible physiopathological mechanisms, and iii) development of new and effective therapeutic strategies. Metabolomics, epigenomics, and proteomics are three of the most common omic technologies used in clinical trials. A variety of processes mediate and change protein functions. Protein phosphorylation, acetylation, methylation, and other post-translational modifications are frequently necessary for functions or signaling. To construct the machinery that performs the requisite cellular tasks, pre-protein folding and post-translational processing as well as the formation of multiprotein complexes are required. Ultimately, whether an expressed protein is active or inactive is often determined by its cellular location. As a result, no single methodology can adequately examine the proteome in all its features, and most proteomics methodologies focus on the identification and quantification of individual proteins, which are commonly carried out using mass spectrometry or affinity-based protein arrays.

Wang-Su et al. reported an abundance of αA-, αB-, and βB2-crystallins in both freshly isolated and cultured human lens epithelial (HLE) cells (Wang-Su et al., 2003). Semba et al. concluded that the ECM composition and homeostasis are altered in the iris in primary closed-angle glaucoma (PACG) through a high-throughput proteomics approach (Semba et al., 2021). The practice of multiomics research in the ocular tissues of neurological disorders and other pathological conditions has gained momentum with advanced technological techniques (Wang-Su et al., 2003; Vogeser and Seger, 2008; Sethi and Zaia, 2017; Bai et al., 2020; Semba et al., 2021). Furthermore, a recent study by Alicia De Maria et al. found that fibrillin-1, latent transforming growth factor-β-binding protein-2, latent transforming growth factor-β-binding protein-3, and lysyl oxidase-like 1 were abundant in the exfoliative material constituent of the human lens capsule specimen by mass spectrometry (2D-LC/MS) analysis. Their research is more focused on the confirmation of the constituents of exfoliative material components present in the eye, but still, the potential signaling roles of these molecules are unanswered (De Maria et al., 2021). It is logical, therefore, that the proteome profile of the tissues underlying the mechanism of glaucoma progression yields further insights into the pathological processes and pathways involved. They can also be useful in deciphering molecular pathways for various eye diseases including glaucoma and in identifying promising protein targets for drug action. Our previous study highlighted different clinical correlates characterizing different phenotypes and disease severity in PXG compared to primary open-angle glaucoma (POAG) and PACG, with evident changes in the tear and aqueous proteome (Sahay et al., 2017). Differential expression of several molecular biomarkers could therefore be utilized as a signal for disease progression in PXF and primary glaucoma (Rao et al., 2021; Sahay et al., 2021). Gene and protein expression studies of glaucoma-associated markers were studied by several groups, indicating their role in the pathogenesis of glaucoma (Margarita et al., 2017; Ayala and Cuklev, 2018). However, there is a lacuna in the differential protein expressions at the ocular surface with the potential signaling roles in different severities of glaucoma (PXF/PXG). We are trying to use the proteomics approach in the different tissues for the identification of biomarkers with the signaling pathways which has not been explored to our knowledge. To gain new insight into PXF pathophysiology, we conducted a hypothesis-free, discovery-phase proteomic investigation using a proteomics approach to characterize alterations in the lens capsule, iris, and trabecular meshwork proteins associated with PXG.

## Materials and Methods

### Clinical Characterization of Cases and the Control With Sample Collection

This study included patients with pseudoexfoliation after a thorough slit lamp examination, Goldmann applanation tonometry, four-mirror gonioscopy, +90D fundus biomicroscopy, and visual fields. Pseudoexfoliation was diagnosed in the presence of exfoliative flaky deposits in the anterior chamber with or without raised IOP. The staging and phenotype classifications followed in the patients included are detailed elsewhere (Rao and Padhy 2014; Sahay et al., 2017; Sahay et al., 2021). Individual patients that fulfilled inclusion criteria and were scheduled for surgery (for cataract or combined cataract + glaucoma) were included in this study after written informed consent. The study followed the principles of the Helsinki Declaration and was approved by the Institute Review Board of the LV Prasad Eye Institute, MTC campus, Bhubaneswar, India.

Non-glaucoma control subjects consisted of individuals who have no history of any ocular disease or abnormality selected from a general eye clinic with no previous history of topical therapy. We also obtained control tissues from the donor eyes harvested from the eye bank or cadaver eyes with no history of glaucoma in patients and their families. Patients with uveitis, neovascular glaucoma, laser or anti-glaucoma therapy, conjunctive disease, allergic blepharitis, or dry eyes were excluded. Patients with autoimmune or neurodegenerative systemic illness or diabetes mellitus or those with evidence of intraocular inflammation were also excluded from this study.

The excised lens capsules were collected before entering the anterior chamber without lens or iris touch, while TM and iris specimens were collected during trabeculectomy. The excised specimens were collected in a sterile Eppendorf tube and stored at −80°C until further experimental assays.

### Proteomic Investigation of *ex vivo* Surgically Dissected Specimens by Label-Free LC-MS/MS Mass Spectrometry

In this study, we analyzed the *ex vivo* dissected lens capsule (LC), iris, and trabecular meshwork (TM) specimens obtained from patients with PXF (*n* = 3), PXG (*n* = 3), and the control (*n* = 3) each during surgical trabeculectomy and from donor corneal buttons obtained from the institutional eye bank for a detailed protein expression profile. The samples were divided as follows: PXG (*n* = 3) was compared to the control (*n* = 3) for LC, PXG (*n* = 3) (the same PXG sample as was used in the comparison with the control) was compared to PXF (*n* = 3) for LC, PXG (*n* = 3) was compared to the control (*n* = 3) for TM, and PXG (*n* = 3) was compared to the control (*n* = 3) for the iris. Each excised tissue was dissolved in 150 ul pre-chilled 6 M Gn-HCL in 0.1 M Tris (pH 8.5) with 1X protease inhibitors (Roche). The tissue was homogenized using a tissue homogenizer and then subjected to probe sonication (15 pulses per 10 s with 10 s between each pulse). Lysed samples were centrifuged (15,000 g for 20 min at 4°C), and the protein in the supernatant was analyzed using the BCA (Thermo) technique. Solubilized protein lysate of Gn-HCL was first reduced using 5 mM TCEP [Tris (2-carboxyethyl) phosphine hydrochloride] with 25 μg of protein lysates, followed by alkylation by 50 mM iodoacetamide. Alkylated proteins were further diluted using 50 mM ammonium bicarbonate to bring the final Gn-HCL concentration to 0.6 M and were then digested for 16 h at 37°C using a 1:50 enzyme to protein ratio using trypsin protease (Promega Gold). The peptide digests were purified by the manufacturer’s procedure using C18 silica cartridges (The Nest Group, Southborough, MA) and finally dried with 50°C speed vacuum. The dried purified peptide pellet was stored at −20°C. Before nano-ESI, the samples were re-suspended in buffer A (5% acetonitrile/0.1% formic acid) to perform LC-MS/MS run.

For label-free spectroscopic analysis of peptide mixtures, the peptide samples have been resolved with an EASY-nLC 1000 (Thermo Fisher Scientific) system, coupled to the QExactive orbitrap mass spectrometer, and equipped with a nanoelectrospray ion source. One microgram of the peptide mixture of each sample was loaded on a 2 cm precolumn (Acclaim Pepmap C18, 3 µM resin) and resolved using an EASY-Spray LC column with 2 µM resin, a 25 cm length, and C18-resin (Thermo Fisher Scientific). The peptides were loaded with buffer A and eluted for 90 min at a flow rate of 250 nl/min by a 0–40% buffer B gradient (95% acetonitrile/0.1% formic acid). Full scan or precursor scan and MS/MS or MS2 scan resolutions of 70,000 and 17,500 at m/z 400, respectively, were implemented for the QExactive run program using a top 10 HCD data-dependent positive polarity acquisition mode. The option of lock mass for poly-dimethyl cyclosiloxane (PCM) ions (m/z = 445.120025) was activated for internal recalibration in a 90-min run. MS data were acquired using a data-dependent top10 method, dynamically choosing the most abundant precursor ions from the survey scan.

#### Database Searching/Quantification and Statistical Analysis

For data analysis, all 21 raw files (three sets each of PXG vs. PXF for LC, PXG vs. control for LC, PXG vs. control for TM, and PXG vs. control for the iris) were analyzed with Proteome Discoverer v2.2 against the SwissProt human reference proteome database (20,366 entries). Mass tolerances were established at 10 ppm and 0.5 Da for MS and MS2 for the SEQUEST search engine. The protease used to generate peptides, that is, enzyme specificity, was set for trypsin/P (cleavage at the C terminus of K/R unless followed by “P”) along with a maximum missed cleavage value of 2. Carbamidomethyl on cysteine as a fixed modification and oxidation of methionine and N-terminal acetylation were considered as variable modifications for database search. Both the peptide spectrum match and protein false discovery rate were set to 0.01 FDR and determined using a percolator node. Relative protein quantification of the proteins was performed using the Minora feature detector node of Proteome Discoverer 2.2 with default settings and considering only high PSM (peptide spectrum matches) confidence. Based on the Uniprot accession number, Pfam, KEGG pathways, and GO annotations were assigned for the list of identified proteins. The data matrix was imported into the Perseus software (version 1.6.0.7). The abundance values of these filtered proteins were log2-transformed, and imputation was applied using Perseus default settings (width 0.3, downshift 1.8), where missing values were replaced with random numbers that are drawn from a normal distribution. Perseus and R scripts were applied between the diseased group samples using a *p*-value significance threshold level of 0.05. The software programs used for the analysis were Thermo Proteome Discoverer (v2.2), STRING (https://string-db.org/), and FunRich (3.1.1). Differentially expressed proteins were assessed, and a fold change threshold was calculated. Identified target proteins (≥2.5) were analyzed for functional enrichment analysis which was obtained through the functional enrichment analysis tool (FunRich software) to delineate specific cellular components, molecular functions, and biological processes involving the proteins identified in the patients with PXF and PXG.

### Immunohistochemistry

Samples of human lens capsules from PXF (*n* = 5), PXG (*n* = 5), and the control (*n* = 5) were harvested from the superior quadrant surgery (before any incision) of patients undergoing either glaucoma or cataract surgery. These samples were analyzed for TGFβ1, procollagen C-endopeptidase enhancer 1 (PCOLCE), anti-alpha smooth muscle actin (α-SMA), fibulin V, and fibronectin (FN1) expressions. The protocol of immunohistochemistry was strictly followed as previously described (Sahay 201b). The sections were incubated with anti-TGFβ1 (1:150, ab27969; Abcam), anti-PCOLCE (4 ug/ml, CL6567; Abcam), anti-a-SMA (1:100, Abcam, ab7817), anti-fibulin V (1:500, ab109428; Abcam), and anti-FN1 (1:50, ab6328; Abcam) at 4°C overnight. After washing with PBS, sections were incubated with the biotinylated secondary goat anti-mouse antibody (Dako) for 30 min. Immune reactions were visualized with incubation in 3, 3′-diaminobenzidine tetrahydrochloride (DAB) for 8 min in the dark following the manufacturer’s protocol (LSAB2 System-HRP, Dako). Sections were counterstained with hematoxylin and fixed subsequently. Slides were examined under a bright-field (CKX53, Olympus, Tokyo, Japan) microscope at ×20 and ×40 magnifications, and images were analyzed by MagVision software.

In each case, the percentage of TGFβ1, PCOLCE, a-SMA, fibulin V, and FN1-positive staining of each slide in triplicates was determined. Tissue sections were scored by determining the proportion of the stained ECM near cells relative to the overall ECMs. Evaluation of TGFβ1, PCOLCE, a-SMA, fibulin V, and FN1 expressions was performed at ×40 magnification. The distribution (%) of each antibody was evaluated according to the following criteria: 0 (<5), 1 (6–25), 2 (25–50), 3 (51–75), and 4 (>75) of cells displaying positive immunoreactivity. The intensity of antibody immunostaining was scored as 0 (none), 1 (weak), 2 (moderate), and 3 (strong). Total immunostaining scores, with combined intensity and distribution of immunostaining, were ranked as low (intensity 0–1 and distribution 0–4 or intensity 2 and distribution 0–1) or high (intensity 2–3 and distribution 2–4) (Sahay et al., 2017; Sahay et al., 2021).

### Cytokine Analysis

The concentration of cytokines in the extracted protein samples from tissues was evaluated using a convenient bioplex kit assay (Milliplex MAP kit, HCYTMAG-60K-PX41, Millipore, Massachusetts, United States), and the protocols were strictly followed as per the manufacturer’s directions. Briefly, surgically excised tissues of LC each from PXF (*n* = 10), PXG (*n* = 10), and the control (*n* = 10) were pooled, whereas tissues of the iris and TM from PXG (*n* = 20) and the control (*n* = 20) were pooled from the same group for each tissue in triplicates and minced into small pieces and homogenized uniformly in 200 ul of the protein extraction buffer (RIPA buffer) using TissueRuptor (QI-AGEN group). Samples were strongly vortexed and heated at 95°C for 5 min and centrifuged at 13,000 rpm for 20 min. The supernatant was collected in a fresh tube and properly marked for the evaluation of different cytokines in each sample. A detailed list of cytokines is given in Supplementary Table S1.

The plates were run on a Bio-Plex^®^ 200 system (Luminex Corporation, Texas, United States). Before each assay run, the system was calibrated with the Bio-Plex^®^ calibration kit and validated with the Bio-Plex^®^ validation kit 4.0. The Bio-Plex^®^ sheath fluid served as the delivery medium for the samples. Analysis was performed with Bio-Plex^®^ manager 6.1, and the software used was xPONENT software. Within the device settings, 50 events per bead region were defined as the minimum criterion.

### Quantification and Statistical Analysis

Perseus and R scripts were applied between the diseased group samples using a *p*-value with a significance threshold level of 0.05 for mass spectrometry analysis. Moreover, data from other experiments, such as IHC and cytokines/chemokines experiments, were analyzed using Graph-Pad Prism with column and grouped comparison. The results were presented as means ± standard error of the mean (SEM) of triplicate experiments. Data were analyzed by the ANOVA post hoc *t*-test, and Tukey correction with a *p*-value of <0.05 is considered to be significant.

## Results

A total of 74 patients were analyzed, including 18 PXF, 28 PXG, and 28 controls. Table 1 shows no statistical difference in the age of the patients between cases (PXF + PXG) and controls (*p*-value = 0.0426). It was noticed that the mean baseline IOP significantly differed between cases and controls, with the maximum IOP seen in PXG cases (*p*-value = 0.0035).

**TABLE 1:** Demographic and clinical characteristics of patients included in the study.

	PXF (*n* = 18)	PXG (*n* = 28)	Control (*n* = 28)	*p*-value
Age (years)	69.14 ± 10.7	71.21 ± 9.99	64.8 ± 7.12	0.0426
Male	72	61	62	0.11
Female	50	64	65	0.12
Baseline IOP	16.892 ± 4.16	28 ± 6.34	13.83 ± 5.45	0.0356

IOP, intraocular pressure; PXF, pseudoexfoliation; PXG, pseudoexfoliation glaucoma.

**FIGURE 1:**
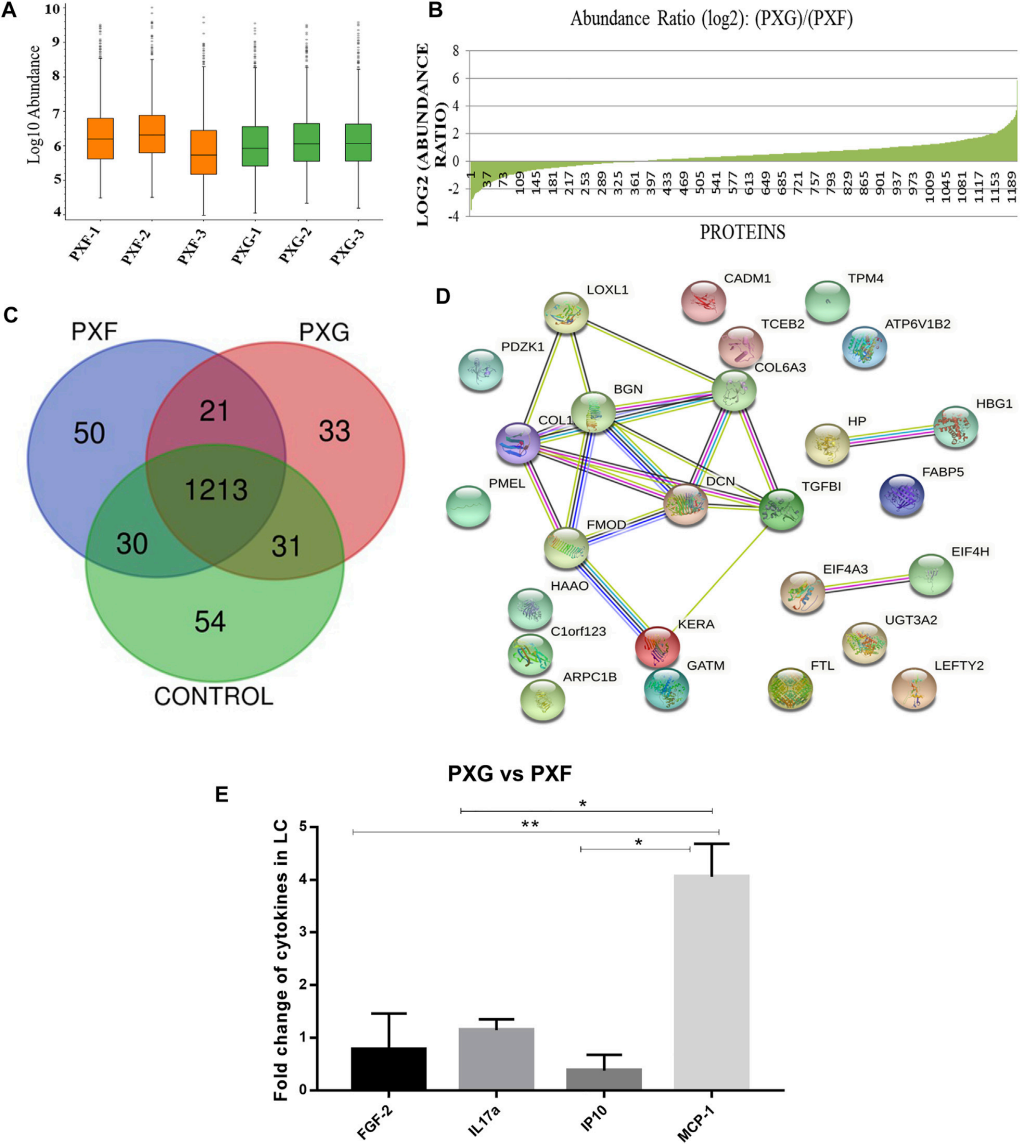
Protein profiles in LC with PXG compared to PXF. **(A)** Box plot of the protein expressions in each sample of PXF and PXG (*n* = 3), showing very little variability among the triplicate samples. **(B)** Abundance ratios (log2) of proteins in PXF vs. PXG with overexpressed or underexpressed proteins indicated in the graph. **(C)** Venn list was derived of all 1,433 proteins in PXF vs. control, PXG vs. control, and PXF vs. PXG for the identification of common and unique proteins in PXF and PXG. **(D)** Network generated of protein–protein interactomes with the top 27 proteins identified in PXG (> 2.5 folds) involved in extracellular homeostasis and protein binding using STRING analysis. **(E)** Fold change of cytokines/chemokines using a bioplex kit assay with PXG compared to PXF. Means ± SEM shown, *p < 0.05, **p < 0.01, one-way ANOVA post hoc *t*-test with Tukey correction, PXF-pseudoexfoliation, and PXG-pseudoexfoliation glaucoma.

Global proteomic analysis of lens capsules from different groups of patients with PXF and PXG was performed using label-free liquid chromatography with tandem mass spectrometry (LC-MS/MS). Figure 1A shows the box plot of the protein expressions in each sample of PXF and PXG, showing very little variability among the samples. Data revealed that 1432 proteins were identified in the lens capsule supported by 13,072 peptides. We found a total of 1202 unique elements (filtered based on master proteins) in each group of PXF and PXG samples (Figure 1B). A Venn diagram was plotted, and a Venn list was derived of all 1433 proteins in three different comparisons as PXF vs. control, PXG vs. control, and most importantly PXF vs. PXG (Figure 1C). A total of 21 proteins were common in PXF and PXG, and the details of the proteins are listed in Table 2. Out of the 1202 proteins, 35 proteins were differentially regulated; among these, the Log2 abundance ratios of 27 proteins were overexpressed (>2.5 fold), and eight proteins were underexpressed (<−2.5 fold) (Table 3). Interestingly, the abundance ratio of lysyl oxidase homolog 1 (LOXL1) was 12.92 times overexpressed in PXG than in PXF, which is responsible for catalyzation in the formation of cross-links in ECM molecules such as collagen or elastin proteins. Protein–protein networks were performed in 27 overexpressed proteins using STRING analysis, depicting predicted functional interactions between proteins involved in ECM homeostasis and protein binding. These highlighted colored nodes represent the overexpressed proteins. LOXL1 (lysyl oxidase homolog 1), BGN (biglycan), DCN (decorin), COL6A3 [collagen alpha-3(VI) chain], COL1A1 [collagen alpha-3(VI) chain], and FMOD (fibromodulin) were shown to have a common point of convergence by transforming growth factor-1 (TGFβ1) (by similarity). These proteins were responsible for fibril formation and collagen fiber assembly (PPI enrichment *p*-value = 1.23e-08) (Figure 1D). Cytokine profiles were also checked by a 41-bioplex kit assay. It was found that MCP production was significantly increased in PXG compared with PXF (*p*-value = 0.0056), whereas EGF-2, IL17a, and IP-10 did not show any change in PXG and PXF samples (Figure 1E).

**TABLE 2:** Common proteins in pseudoexfoliation (PXF) syndrome and pseudoexfoliation glaucoma (PXG).

Sl. No	Accession number	Protein name
1	P40123	Adenylyl cyclase-associated protein (CAP1)
2	P02724	Glycophorin-A (GYPA)
3	P01700	Ig lambda chain V-I region HA
4	Q96DR8	Mucin-like protein 1 (MUCL1)
5	P36543	V-type proton ATPase subunit E 1 (ATP6V1E1)
6	Q92896	Golgi apparatus protein 1 (GLG1)
7	Q07507	Dermatopontin (DPT)
8	P43121	Cell surface glycoprotein MUC18 (MCAM)
9	P04433	Ig kappa chain V-III region VG (Fragment)
10	P37840	Alpha-synuclein OS = *Homo sapiens* (SNCA)
11	P55774	C-C motif chemokine 18 (CCL18)
12	P06727	Apolipoprotein A-IV (APOA4)
13	P01877	Ig alpha-1 chain C region (IGHA1)
14	Q06828	Fibromodulin (FMOD)
15	P02747	Complement C1q subcomponent subunit C (C1QC)
16	P01036	Cystatin-S (CST4)
17	P20930	Filaggrin (FLG)
18	O95361	Tripartite motif-containing protein 16 (TRIM16)
19	Q9GZS3	WD repeat-containing protein 61 (WDR61)
20	P56381	ATP synthase subunit epsilon, mitochondrial (ATP5E)
21	P20774	Mimecan OS = *Homo sapiens* (OGN)

**TABLE 3:** Top overexpressed and underexpressed proteins in the lens capsules with pseudoexfoliation glaucoma (PXG) compared to pseudoexfoliation (PXF) syndrome.

Overexpressed proteins >2.5 folds			
Sl no	Accession number	Protein name	Abundance ratio Log2 (PXG v/s PXF)
1	P40967	Melanocyte protein (PMEL)	2.5164977
2	P67936	Tropomyosin alpha-4 chain (TPM4)	2.5445201
3	P46952	3-hydroxyanthranilate 3,4-dioxygenase (HAAO)	2.5941029
4	Q15056	Eukaryotic translation initiation factor 4H (EIF4H)	2.5995809
5	P12111	Collagen alpha-3(VI) chain (COL6A3)	2.6328845
6	O15143	Actin-related protein 2/3 complex subunit 1B (ARPC1B)	2.6425627
7	P38919	Eukaryotic initiation factor 4A-III (EIF4A3)	2.6603905
8	P50440	Glycine amidinotransferase, mitochondrial (GATM)	2.7508166
9	P30711	Glutathione S-transferase theta-1 (GSTT1)	2.7638696
10	Q06828	Fibromodulin (FMOD)	2.7716544
11	Q3SY77	UDP-glucuronosyltransferase 3A2 (UGT3A2)	2.8206772
12	Q15370	Transcription elongation factor B polypeptide 2 (TCEB2)	2.828358
13	P21281	V-type proton ATPase subunit B, brain isoform (ATP6V1B2)	2.9473999
14	O00292	Left-right determination factor 2 (LEFTY2)	2.9552869
15	Q9NWV4	UPF0587 protein C1orf123 (C1orf123)	2.9785397
16	Q15582	Transforming growth factor-beta-induced protein ig-h3 (TGFBI)	2.9803986
17	Q9BY67	Cell adhesion molecule 1 (CADM1)	3.0066145
18	P02452	Collagen alpha-1(I) chain (COL1A1)	3.1023333
19	O60938	Keratocan (KERA)	3.1031179
20	P07585	Decorin (DCN)	3.1678653
21	Q01469	Fatty acid-binding protein, epidermal (FABP5)	3.2128678
22	P00738	Haptoglobin (HP)	3.2729405
23	P21810	Biglycan (BGN)	3.3391596
24	Q5T2W1	Na(+)/H(+) exchange regulatory cofactor NHE-RF3 (PDZK1)	3.6686654
25	Q08397	Lysyl oxidase homolog 1 (LOXL1)	3.6922752
26	P02792	Ferritin light chain (FTL)	3.8773139
27	P69891	Hemoglobin subunit gamma-1 (HBG1)	5.8273712
**Underexpressed proteins <−2.5 folds**			
1	O75251	NADH dehydrogenase [ubiquinone] iron-sulfur protein 7, (NDUFS7)	−3.5050891
2	P20774	Mimecan (OGN)	−3.4995671
3	P07315	Gamma-crystallin C (CRYGC)	−3.4663718
5	O60664	Perilipin-3 (PLIN3)	−2.7495566
6	Q15262	Receptor-type tyrosine-protein phosphatase kappa (PTPRK)	−2.6854224
7	P01040	Cystatin-A (CSTA)	−2.6790833
8	Q00796	Sorbitol dehydrogenase (SORD)	−2.5792797

PXG, pseudoexfoliation glaucoma; PXF, pseudoexfoliation syndrome.

**FIGURE 2:**
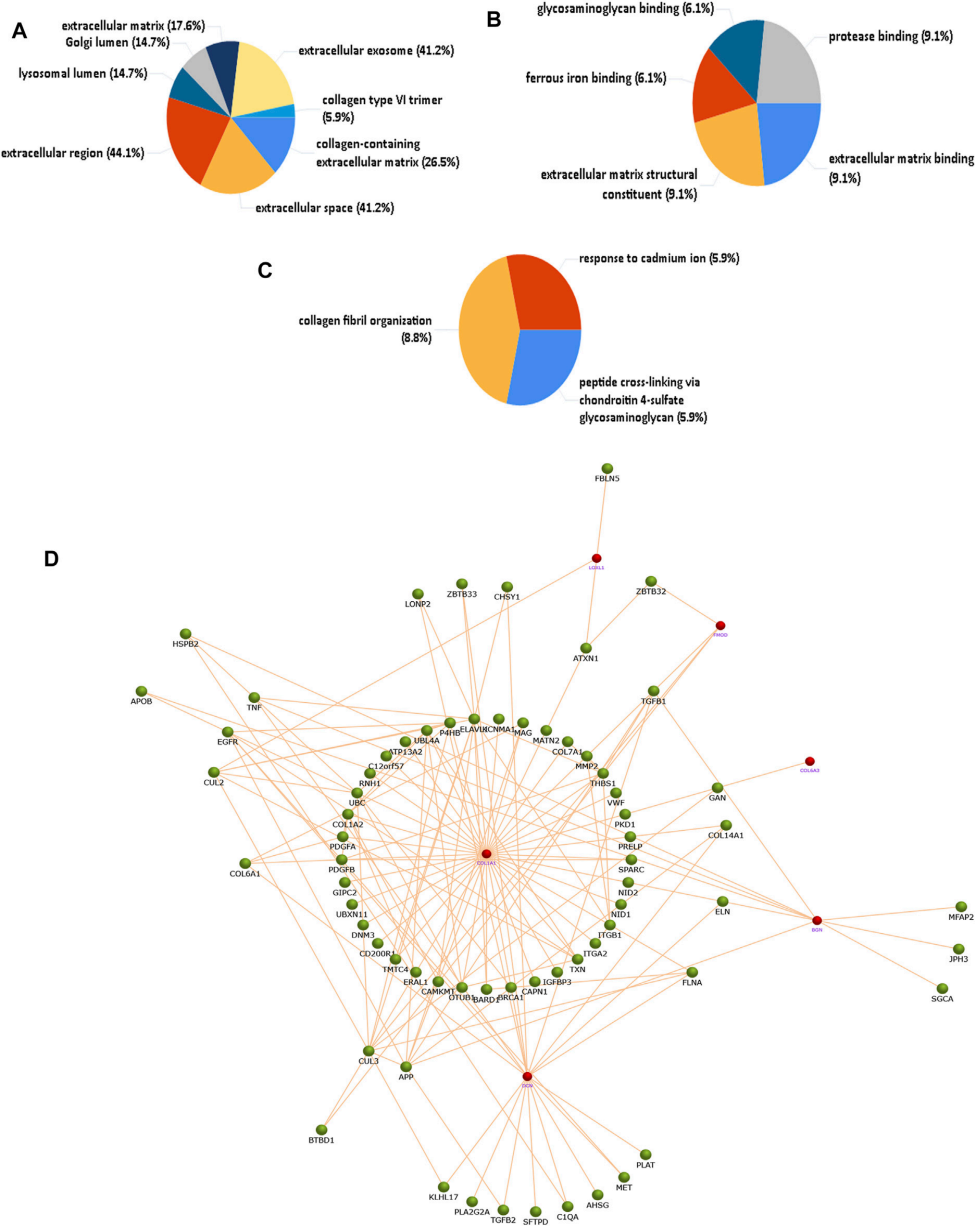
Functional enrichment protein analysis of overexpressed/underexpressed PXG proteins compared to PXF by the FunRich database tool. A total of 27 overexpressed and eight underexpressed proteins were subjected to this analysis. **(A)** Percentage of the cellular component showing the collagen type-VI trimer more enriched in PXG. **(B)** Percentage of molecular functions showing glycosaminoglycan binding, ferrous iron binding, and ECM binding more enriched in PXG. **(C)** Percentage of biological process classification showing peptide cross-linking, collagen fibril organization, and response to cadmium ions more enriched in PXG. **(D)** Protein interaction of neighboring proteins from the UniProt dataset involved in four major pathways showing maximum protein counts including “ECM proteoglycans,” “assembly of collagen fibrils and other multimeric structures,” “collagen biosynthesis and modifying enzymes,” and “cross-linking of collagen fibrils” by six important proteins (Col1A1, Col6A3, LOXL1, FMOD, DCN, and BGN). The red node represents the target protein, and the green node represents the neighboring protein. Col1A1-collagen alpha-1(I) chain, Col6A3-collagen alpha-3(VI) chain, LOXL1-lysyl oxidase homolog 1, FMOD-, DCN-decorin, and BGN-biglycan.

The identified proteins described in Table 3 which were overexpressed/underexpressed were subjected to functional enrichment protein analysis through the FunRich database tool (3.1.1). Figures 2A–C shows the results, sorted by percentage of the cellular component, molecular function, and biological process classification obtained through FunRich investigation in PXG patients as compared to PXF. Data showed that “collagen type-VI trimer” was 365.6 times more enriched as a cellular component in PXG in respect to PXF, which corresponds to only 5.9% (*p*-value = 0.004), whereas the molecular functions such as glycosaminoglycan binding, ferrous iron binding, and extracellular matrix binding are 37.1 (*p*-value = 0.007), 38.9 (*p*-value = 0.006), and 49.8 (*p*-value = 0.004) times, respectively, more enriched in PXG than in PXF patients. The enrichment of biological functions such as “peptide cross-linking” is 165.3 (*p*-value = 0.05), “collagen fibril organization” is 27 (*p*-value = 0.006), and “response to cadmium ion” is 36.8 times (*p*-value = 0.007) found to be highlighted in PXG compared to PXF patients. We performed functional protein network analyses on the differentially expressed proteins to understand the molecular mechanisms and biological processes that are altered in the eye in response to exposure to increased IOP. We identified that the pathways indicating ECM proteoglycans, assembly of collagen fibrils and other multimeric structures, collagen biosynthesis and modifying enzymes, and cross-linking of collagen fibrils were highlighted, showing a maximum of protein counts (*p*-value = 0.004). Figure 2D reveals the interaction of neighboring proteins from the UniProt data set, which is involved in these four major pathways by six important proteins (Col1A1, Col6A3, LOXL1, FMOD, BGN, and DCN from the Table 3 datasheet) identified by the software.

**FIGURE 3:**
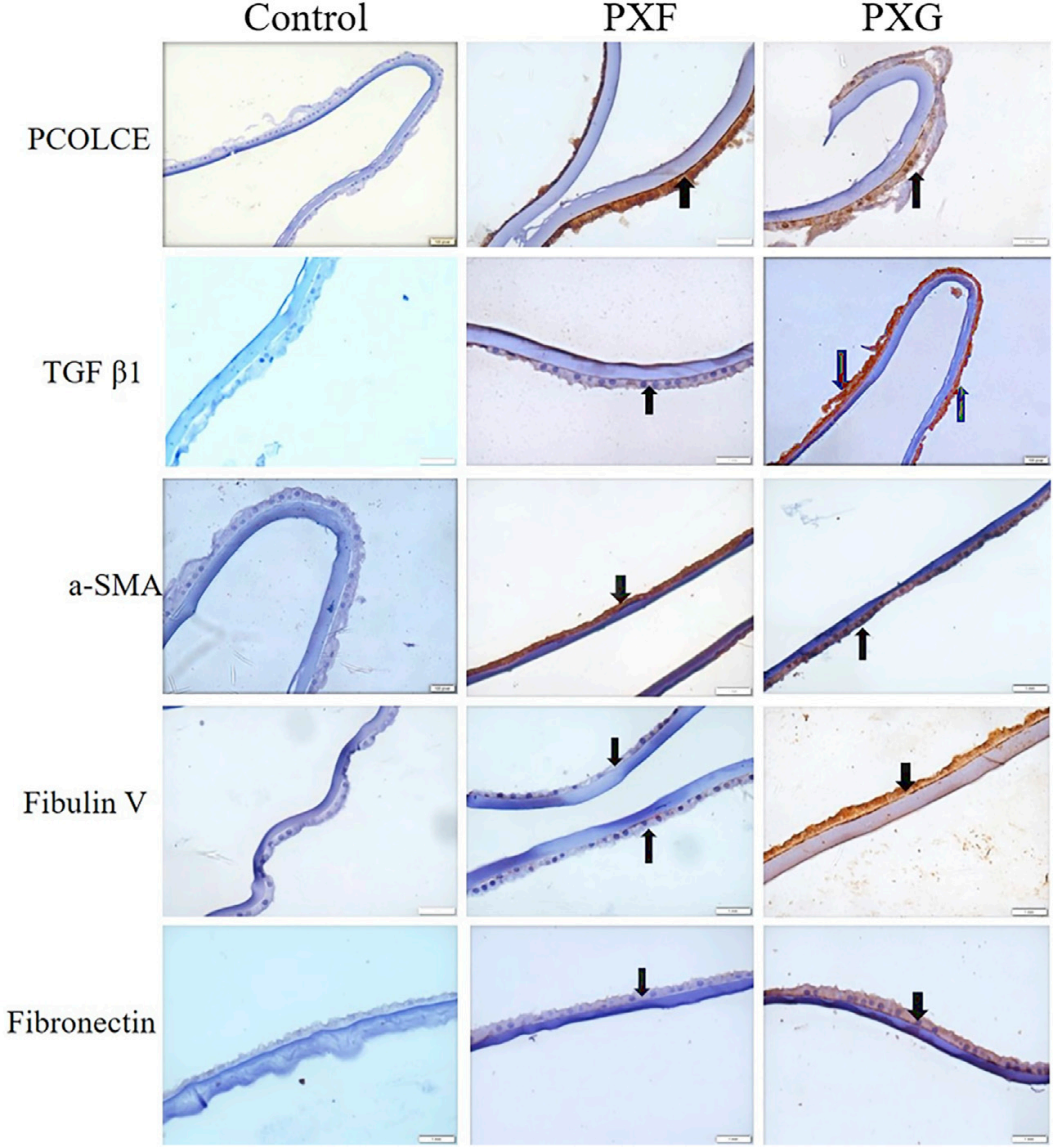
Evaluation of the protein of interest involved in ECM aggregation in LCs by immunohistochemistry. Bright-field microscopic images at ×40 magnifications of PCOLCE, TGFβ1, a-SMA, fibulin V, and fibronectin in the LC. Black arrows indicate immunopositivity of the highlighted/target proteins in the LC. TGFβ1, a-SMA, fibulin V, and fibronectin were highly expressed in PXG compared to PXF/control. Scale bar-10 um, PXF-pseudoexfoliation, PXG-pseudoexfoliation glaucoma, PCOLCE-procollagen C-endopeptidase enhancer 1, TGFβ1-transforming growth factor, a-SMA (anti-alpha smooth muscle actin).

Immunohistochemistry of the lens capsule for the expression of TGFβ1, PCOLCE, α-SMA, Fib V, and FN1 and proteins involved in ECM aggregation showed overexpression of these markers maximally in PXG and then in PXF compared to controls. We found protein expression of PCOLCLE and α-SMA in the cells of the epithelial layer and cytoplasm of the lens epithelium with PXF and PXG. Fibulin-V and TGFβ-1 expressions were less in the cytoplasm in PXF, but their expressions were increased in the cells of the epithelial layer and cytoplasm of the lens epithelium of LC with PXG. FN1 was more expressed in the cytoplasm of the lens epithelium of LC in PXG than in PXF (Figure 3, Supplementary Table S2). The concurrent increases in expression of both LOXL1 (as per proteomics data) and FN1 are due to a variety of reasons. *In vitro*, oxidative stress, hypoxia, and increased levels of pro-fibrotic cytokines, such as interleukin (IL)-6, and growth factors, such as TGFβ-1, have been demonstrated to regulate (i) LOXL1 and clusterin expression as well as (ii) matrix molecule synthesis, including elastin and FN1. TGFβ-1 and oxidative stress have both been shown to have a major impact on the coordinated expression of LOXL1 and FN1 (Klingberg et al., 2013). Although TGFβ can directly stimulate aSMA expression, there are other indirect mechanisms and interactions with other TGFβ-induced proteins to consider (Nagamoto et al., 2000).

**FIGURE 4:**
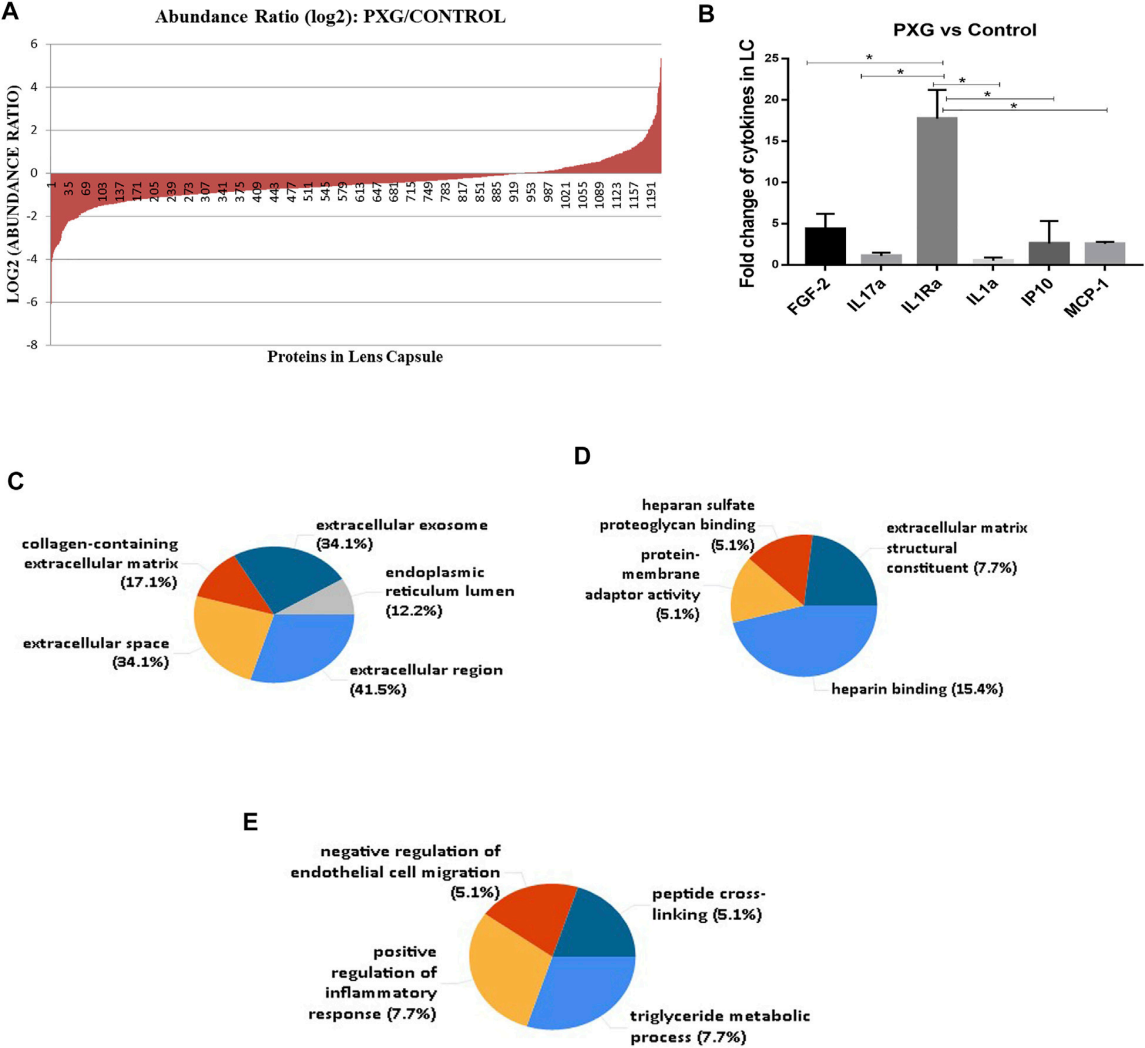
Protein profile in LC with PXG compared to the control. **(A)** Abundance ratios (log2) of proteins overexpressed or underexpressed are indicated in the graph. **(B)** Fold change of cytokines/chemokines using a bioplex kit assay with PXG compared to the control. **(C)** Functional enrichment protein analysis by the FunRich database tool using 43 proteins subjected to this analysis. The percentage of the cellular component highlights chylomicron more enriched in PXG. **(D)** Molecular functions such as heparan sulfate proteoglycan binding and protein-membrane adaptor activity are more enriched in PXG. **(E)** Biological process classifications such as peptide cross-linking are more enriched in PXG. Means ± SEM shown, *p < 0.05, **p < 0.01, ***p < 0.001, ****p < 0.0001, one-way ANOVA post hoc *t*-test with Tukey correction, PXG-pseudoexfoliation glaucoma.

We have also compared the protein profile of PXG samples with that of control samples in LC. Here, we observed that a total of 1,211 proteins supported by 13,072 peptides were identified, out of which we found 1,209 unique elements (filtered based on master proteins) in each group of PXG and control samples (Figure 4A). In the group of 1,209 proteins, 43 proteins were differentially regulated; among these, 15 proteins were overexpressed (≥2.5 fold) and 26 proteins were underexpressed (≤−2.5 fold) (Table 4). Cytokines were also checked in the PXG sample and compared with those in the control. We found that IL1Ra, FGF-2, and IP-10 were significantly upregulated in PXG when compared to the control (*p*-value = 0.0009), whereas IL17a, IL1a, and MCP-1 did not show any change in PXG (Figure 4B). Figures 4C–E demonstrate the results of the FunRich investigation in PXG patients against controls, organized by the percentage of the cellular component, molecular function, and biological process classification. Data revealed that “chylomicron” is 70.1 times more enriched as a cellular component, whereas the molecular functions such as “heparan sulfate proteoglycan binding” and “protein-membrane adaptor activity” are 45.3 and 45.5 times, respectively, more enriched in PXG than in the control. The enrichment of biological functions such as “peptide cross-linking” is 30 times more in PXG than in the control.

**TABLE 4:** Top overexpressed and underexpressed proteins in the lens capsules with pseudoexfoliation glaucoma (PXG) compared to the control.

Overexpressed proteins >2.5 folds			
Sl no	Accession number	Protein name	Abundance ratio Log2 (PXG v/s control)
1	P06858	Lipoprotein lipase	2.5345854
2	P07203	Glutathione peroxidase 1	2.5383394
3	P01040	Cystatin-A	2.5877389
4	P07585	Decorin	2.6818904
5	P02749	Beta-2-glycoprotein 1	2.7238997
6	P07315	Gamma-crystallin C	2.8072435
7	P08603	Complement factor H	2.9453998
8	Q9ULM2	Zinc finger protein 490	3.1768965
9	Q86YZ3	Hornerin	3.7453
10	Q15262	Receptor-type tyrosine-protein phosphatase kappa	3.9638694
11	P12277	Creatine kinase B-type	4.0474168
12	O75888	Tumor necrosis factor ligand superfamily member 13	4.0548092
13	O76041	Nebulette	4.2238006
14	O60938	Keratocan	4.9356438
15	P08123	Collagen alpha-2(I) chain	5.3362677
**Underexpressed proteins <−2.5 folds**			
1	P02792	Ferritin light chain	−6.0666146
2	Q9Y2U8	Inner nuclear membrane protein Man1	−4.363643
3	P21980	Protein-glutamine gamma-glutamyltransferase 2	−4.1034401
4	Q5TDP6	Lengsint test	−3.9245978
5	Q5T2W1	Na(+)/H(+) exchange regulatory cofactor NHE-RF3	−3.6721226
6	Q9BPW8	Protein NipSnap homolog 1	−3.5778253
7	Q9UKY7	Protein CDV3 homolog	−3.4907015
8	Q12905	Interleukin enhancer-binding factor 2	−3.4829674
9	P67936	Tropomyosin alpha-4 chain	−3.4573066
10	Q9NYL9	Tropomodulin-3	−3.4127752
11	Q7RTY7	Ovochymase-1	−3.3662561
12	P32320	Cytidine deaminase	−3.3597049
13	P38919	Eukaryotic initiation factor 4A-III	−3.3253106
14	Q7Z4H8	KDEL motif-containing protein 2	−3.3104519
15	Q9BY67	Cell adhesion molecule 1	−3.2564559
16	Q92765	Secreted frizzled-related protein 3	−3.2165498
17	Q9HCB6	Spondin-1	−3.1830843
18	Q2I0M5	R-spondin-4	−3.1290258
19	P07942	Laminin subunit beta-1	−3.0227036
20	P03973	Antileukoproteinase	−2.8654764
21	O94973	AP-2 complex subunit alpha-2	−2.8063827
22	P49765	Vascular endothelial growth factor B	−2.6902622
23	P47985	Cytochrome b-c1 complex subunit Rieske, mitochondrial	−2.6696009
24	P84090	Enhancer of rudimentary homolog	−2.6327673
25	Q9NYU2	UDP-glucose: glycoprotein glucosyltransferase 1	−2.5580759
26	P21741	Midkine	−2.5489301

PXG, pseudoexfoliation glaucoma.

Furthermore, we have also evaluated the protein profile in the iris and trabecular meshwork (TM). In the iris, a total of 1,300 proteins (filtered based on master proteins) supported by 12,133 peptides were identified in each group of PXG and control samples (Supplementary Figure S1A). Out of the 1,300 proteins, 44 proteins were differentially regulated; among these, six proteins were overexpressed (≥2.5 fold) and 38 proteins were underexpressed (≤−2.5 fold) (Supplementary Table S3). Here in this table, we found that beta crystallin S and beta crystallin B2 were overexpressed in PXG. Crystallins may act as critical modulators in glaucoma and thus be integral to the process of glaucomatous neurodegeneration. We also found that cytokines such as GCSF, IFN-a2, PDGF-AA, IL-13, IL-8, IP-10, and MCP-1 were significantly increased in PXG compared with the control (*p*-value= < 0.0001), whereas IL17a, IL1Ra, IL1a, IL2, MIP1b, and TNFα did not show any change in PXG (Supplementary Figure S1B).

In TM, a total of 862 proteins supported by 8,463 peptides were identified, out of which we found 657 unique elements (filtered based on master proteins) in each group of PXG and control samples (Supplementary Figure S1C). In the group of 657 proteins, 137 proteins were differentially regulated; among these, 66 proteins were overexpressed (≥ 2.5 fold) and 71 proteins were underexpressed (≤−2.5 fold) (Supplementary Table S4). Beta crystallin B2, beta crystallin A3, gamma crystallin D, and beta crystallin S were found to be overexpressed in PXG. Cytokines were also checked in the PXG sample compared with the control. We found that MDC, IL1Ra, IP-10, MCP-1, and TNFα were significantly increased in PXG compared to the control (*p*-value = 0.0001), whereas EGF-2, IL17a, IL1a, and IL-2 did not show any change in PXG (Supplementary Figure S1D).

## Discussion

PXF and PXG are clinically significant ECM ailments defined by the pathologic buildup of an aberrant fibrillar substance in various intraocular and extraocular tissues. Its exact etiopathogenesis is yet unknown. According to the studies on the molecular level, PXF is an elastic microfibrillopathy linked with the excessive synthesis of elastic microfibrillar components (Moali et al., 2005; Margarita et al., 2017).

In our study, we conducted label-free LC-MS/MS mass spectrometry on the *ex vivo* dissected lens capsule (LC), iris, and trabecular meshwork (TM) specimens obtained from patients with PXF, PXG, and control samples. A total of 27 proteins were overexpressed, in which LOXL1 is 12.92 times increased in PXG compared with PXF. LOXL1 is the most studied ECM protein that plays a major role in fibrosis. Aberrantly overexpressed LOXL1 might contribute to the development of PXG. We saw that most of the overexpressed proteins involved in collagen fibril assembly and their aggregation result in ECM accumulation.

We also validated the proteins responsible for accumulation and aggregation of ECM such as PCOLCE, TGFβ1, α-SMA, fibulin-V, and fibronectin (FN1) in the tissues of LC through immunohistochemistry. Immunolabeling revealed that PCOLCLE and α-SMA protein expression were high in the cells of the epithelial layer and cytoplasm of the lens epithelium with PXF and PXG. It has been reported that PCOLCE promotes the activity of metalloproteinases that remove C-propeptides from the ECM’s pro-collagen I, II, and III, resulting in collagen deposition (Soares et al., 2020). Increased collagen deposition is widely recognized as the most well-known ECM alteration. α-SMA is a marker of epithelial to mesenchymal transition (EMT). EMT is normally involved in tissue repair, but altered EMT has been shown to play a major role in fibrosis. EMT is pivotal in varying diseases such as lung and kidney fibrosis and cancers (O’reilly et al., 2011). Increased α-SMA in the LC of PXF and PXG hints at the fibroblast to myofibroblast change. Myo-fibroblasts formed due to EMT produce elevated levels of ECM, leading to fibrosis. Fibulin-V was less expressed in the cytoplasm in PXF, but its expressions were high in the epithelial layer and cytoplasm of the lens epithelium of LC with PXG. A similar trend was seen for fibronectin (FN1), where it was more expressed in the cytoplasm of the lens epithelium of LC in PXG than in PXF. FN1 is an important component of the ECM. Cellular fibronectin is an insoluble isoform that forms a fibril network and regulates ECM–cell interactions (Faralli et al., 2019). An increased fibronectin fibril density is thought to increase IOP by altering the compliance of the trabecular meshwork. Recent research suggests that the composition and organization of fibronectin fibrils affect IOP by altering the cell-matrix signaling events that control the functional properties of the cells in the TM (Zhao et al., 2004). Fibulin-V is another ECM protein that plays a role in elastic fiber formation. Both fibronectin and fibulin-V have pro-fibrotic effects and were found to be elevated in PXG. In our previous study, TGFβ1 is elevated in the aqueous humor and serum of PXF and PXG patients (Sahay et al., 2021). These investigations are in parallel with our previous findings and reveal elevated TGFβ1 in the epithelial layer as well as in the cytoplasm of the lens epithelium of LC in PXG patients. TGFβ1 is a major modulator of ECM formation (O’reilly et al., 2011). TGFβ has been shown to promote the phagocytosis of bovine trabecular meshwork cells *in vitro* Cao et al., 2001). Additionally, TGFβ1 induces epithelial-mesenchymal transformation in human TM cells in cell culture, which was dose-dependent. TGFβ 1 and 2 are also known to cause ECM production and reduce degradation in cell culture and anterior segment cultures (Koch et al., 2004). It is also known to induce the expression of collagen IV and VI, the connective tissue growth factor, and thrombospondin while inhibiting MMPs and other ECM degradation pathways (Gonzalez et al., 2000; Koch et al., 2004).

A few studies in proteome profiles of the aqueous humor have also been extensively studied. For example, a recent study by Liu et al. explored the differential proteome in the aqueous humor (AH) with different primary glaucomas such as primary acute angle-closure glaucoma (PAACG), primary chronic angle-closure glaucoma (PCACG), and neovascular glaucoma (NVG). Their study highlights that lipid metabolism, immune response, and cell death were significantly activated in glaucoma relative to cataract, and these functions showed different degrees of disorder among the three types of glaucoma (Liu et al., 2021). Botling Taube A et al. studied AH in eyes with PXF, and their group found that the concentration of complement factor 3, kininogen-1, antithrombin III, and vitamin D-binding protein was increased in all eyes with PXF, whereas beta crystalline B1, CRYBB2, and gamma-crystalline D were up to eightfold upregulated in 4 of 10 in eyes with PXF (Botling Taube et al., 2019). Our group is more focused on the proteins involved in the ECM aggregation and accumulation in the lens capsule of the patients with PXG, which is known for the accumulation of protein aggregates. Through different functional annotation and protein interaction networks, we observed the ECM remodeling event playing a potential role and how these proteins could be used as novel targets for glaucoma.

In summary, our results identified changes in the protein profile and cytokine concentrations in different ocular tissues in human patients with PXF and PXG. Our study contributes to the collection of knowledge regarding the etiopathogenetic mechanisms of PXG by providing detailed information on proteins related to glaucoma pathogenesis. Overall, this study would elaborate on important proteins or markers that could serve as a potential target for ECM remodeling and inflammation in glaucomatous eyes and make these proteins a novel target for glaucoma treatment.

## Data Availability

The datasets presented in this study can be found in online repositories. The names of the repository/repositories and accession number(s) can be found below at: http://www.proteomexchange.org/, PXD032202.
