# Supplementary figures and images for: Global and Comparative Proteome Signatures in the Lens Capsule, Trabecular Meshwork, and Iris of Patients With Pseudoexfoliation Glaucoma
Source: Front Mol Biosci. 2022 Apr 20;9:877250. doi: 10.3389/fmolb.2022.877250 (PMC9065473; doi:10.3389/fmolb.2022.877250)

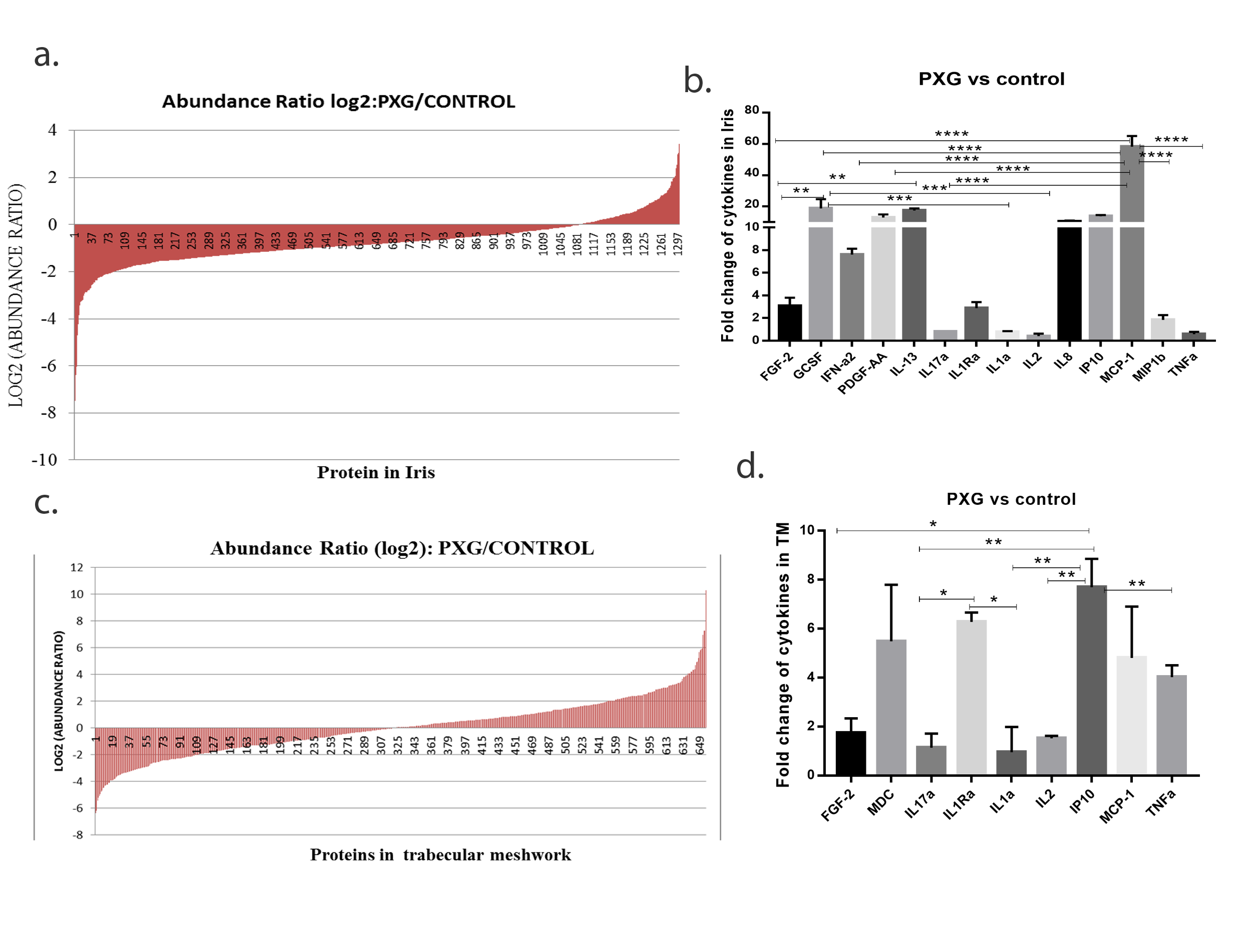

Supplement: Supplementary file 1 [file Image1.TIF]
