# Supplementary material for: Global and Comparative Proteome Signatures in the Lens Capsule, Trabecular Meshwork, and Iris of Patients With Pseudoexfoliation Glaucoma
Source: Front Mol Biosci. 2022 Apr 20;9:877250. doi: 10.3389/fmolb.2022.877250 (PMC9065473; doi:10.3389/fmolb.2022.877250)
Supplement: Supplementary file 2 [file Table1.DOC]

**Table S1.** Full forms of abbreviated cytokines used in bioplex kit assay.

| Scd40L | Soluble CD40-ligand |
| --- | --- |
| EGF | Epidermal Growth Factor |
| FGF-2 | Fibroblast Growth Factor |
| Flt-3 ligand | Fms-related tyrosine kinase 3 ligand |
| G-CSF | Granulocyte colony stimulating factor |
| GM-CSF | Granulocyte Macrophage Colony Stimulating Factor |
| CXCL1/GRO | Growth regulated alpha protein |
| IFN-α2 | Interferon- α2 |
| IFN-γ | Interferon-γ |
| IL-1α | Interleukin-1α |
| IL-1β | Interleukin-1β |
| IL-1ra | Interleukin-1 receptor antagonist |
| IL-2 | Interleukin-2 |
| IL-3 | Interleukin-3 |
| IL-4 | Interleukin-4 |
| IL-5 | Interleukin-5 |
| IL-6 | Interleukin-6 |
| IL-7 | Interleukin-7 |
| IL-8 | Interleukin-8 |
| IL-9 | Interleukin-9 |
| IL-10 | Interleukin-10 |
| IL-12 (p40) | Interleukin-12p40 |
| IL-12 (p70) | Interleukin-12p70 |
| IL-13 | Inteleukin-13 |
| IL-15 | Interleukin-15 |
| IL-17A | Interleukin-17A |
| IP-10 | Interferon- γ produced protein-10 |
| MCP-1 | Monocyte Chemoattractant Protein-1 |
| MCP-3 | Monocyte Chemoattractant Protein-3 |
| MDC | Macrophage Derived Chemokine |
| MIP-1α | Macrophage Inflammatory Protein-1α |
| MIP-1β | Macrophage Inflammatory Protein-1β |
| PDGF-AA | Platelet Derived Growth Factor-AA |
| PDGF-AB/BB | Platelet Derived Growth Factor-AB/BB |
| TGF-α | Transforming Growth Factor-α |
| TNF-α | Tumour Necrosis Factor-α |
| TNF-β | Tumour Necrosis Factor-β |
| VEGF | Vascular Endothelial Growth Factor |
