# Supplementary material for: Global and Comparative Proteome Signatures in the Lens Capsule, Trabecular Meshwork, and Iris of Patients With Pseudoexfoliation Glaucoma
Source: Front Mol Biosci. 2022 Apr 20;9:877250. doi: 10.3389/fmolb.2022.877250 (PMC9065473; doi:10.3389/fmolb.2022.877250)
Supplement: Supplementary file 3 [file Table2.DOCX]

**Table S2.** The immunostaining scores of proteins in lens capsule.

|  | | **Control** | **Pseudoexfoliation (PXF)** | **Pseudoexfolation glaucoma (PXG)** |
| --- | --- | --- | --- | --- |
| **Distribution**  **of (%)** | PCOLCE | 1(6-25%) | 4(>75%) | 4(>75%) |
|  | TGF-β1 | 1(6-25%) | 2(26-50%) | 4(>75%) |
|  | α-SMA | 1(6-25%) | 4(>75%) | 4(>75%) |
|  | Fibulin-V | 1(6-25%) | 1(6-25%) | 4(>75%) |
|  | FN1 | 1(6-25%) | 1(6-25%) | 4(>75%) |
| **Intensity** | PCOLCE | 0 | 3 | 3 |
|  | TGF-β1 | 0 | 1 | 3 |
|  | α-SMA | 1 | 3 | 3 |
|  | Fibulin-V | 0 | 1 | 3 |
|  | FN1 | 0 | 1 | 3 |
| **Total immunostaining scores** | PCOLCE | low | high | high |
|  | TGF-β1 | low | low | high |
|  | α-SMA | low | high | high |
|  | Fibulin-V | low | low | high |
|  | FN1 | low | low | high |
