# Supplementary material for: Global and Comparative Proteome Signatures in the Lens Capsule, Trabecular Meshwork, and Iris of Patients With Pseudoexfoliation Glaucoma
Source: Front Mol Biosci. 2022 Apr 20;9:877250. doi: 10.3389/fmolb.2022.877250 (PMC9065473; doi:10.3389/fmolb.2022.877250)
Supplement: Supplementary file 4 [file Table3.DOCX]

**Table S3.** Top over-expressed and under-expressed proteins in Iris with pseudoexfoliation glaucoma (PXG) compared to control.

| **Over-expressed proteins > 2.5 folds** | | | |
| --- | --- | --- | --- |
| **Sl no.** | **Accession number** | **Proteins name** | **Abundance ratio Log2**  **(PXG v/s Control)** |
| 1 | P05813 | Beta-crystallin A3 | 2.5472363 |
| 2 | P02489 | Alpha-crystallin A chain OS=Homo sapiens | 2.8146561 |
| 3 | Q92945 | Far upstream element-binding protein 2 | 2.970874 |
| 4 | Q9P2T1 | GMP reductase 2 | 3.0180522 |
| 5 | P22914 | Beta-crystallin S | 3.0285149 |
| 6 | P43320 | Beta-crystallin B2 | 3.4136798 |
| **Under-expressed proteins <-2.5 folds** | | | |
| 1 | O43167 | Zinc finger and BTB domain-containing protein 24 | -7.473552 |
| 2 | P13646 | Keratin, type I cytoskeletal 13 | -6.908645 |
| 3 | P02808 | Statherin OS=Homo sapiens | -6.386607 |
| 4 | Q6ZVX7 | F-box only protein 50 | -6.049452 |
| 5 | P19013 | Keratin, type II cytoskeletal 4 | -4.988744 |
| 6 | Q16890 | Tumor protein D53 | -4.715476 |
| 7 | Q9NQB0 | Transcription factor 7-like 2 | -4.505378 |
| 8 | P0C0L5 | Complement C4-B | -4.232479 |
| 9 | P29762 | Cellular retinoic acid-binding protein 1 | -3.841891 |
| 10 | P49789 | Bis(5'-adenosyl)-triphosphatase | -3.577752 |
| 11 | P20933 | N(4)-(beta-N-acetylglucosaminyl)-L-asparaginase | -3.43874 |
| 12 | P35573 | Glycogen debranching enzyme | -3.342983 |
| 13 | P30838 | Aldehyde dehydrogenase, dimeric NADP-preferring | -3.290417 |
| 14 | P01008 | Antithrombin-III | -3.267962 |
| 15 | Q15257 | Serine/threonine-protein phosphatase 2A activator | -3.23026 |
| 16 | P01011 | Alpha-1-antichymotrypsin | -3.226875 |
| 17 | Q16678 | Cytochrome P450 1B1 | -3.222079 |
| 18 | P30711 | Glutathione S-transferase theta-1 | -3.19255 |
| 19 | P62910 | 60S ribosomal protein L32 | -3.167983 |
| 20 | P54136 | Arginine--tRNA ligase, cytoplasmic | -3.027183 |
| 21 | Q86YZ3 | Hornerin | -2.920725 |
| 22 | O43556 | Epsilon-sarcoglycan | -2.879262 |
| 23 | Q9UJC5 | SH3 domain-binding glutamic acid-rich-like protein 2 | -2.870019 |
| 24 | Q92841 | Probable ATP-dependent RNA helicase DDX17 | -2.868428 |
| 25 | Q9Y2V2 | Calcium-regulated heat-stable protein 1 | -2.864725 |
| 26 | Q969P0 | Immunoglobulin superfamily member 8 | -2.86127 |
| 26 | Q00325 | Phosphate carrier protein, mitochondrial | -2.832384 |
| 27 | O00757 | Fructose-1,6-bisphosphatase isozyme 2 | -2.828012 |
| 28 | Q969H8 | Myeloid-derived growth factor | -2.806227 |
| 29 | P20700 | Lamin-B1 | -2.795419 |
| 30 | P05997 | Collagen alpha-2(V) chain | -2.781221 |
| 31 | O95777 | U6 snRNA-associated Sm-like protein LSm8 | -2.767326 |
| 32 | Q5VU65 | Nuclear pore membrane glycoprotein 210-like | -2.73125 |
| 33 | P48539 | Purkinje cell protein 4 | -2.697944 |
| 34 | P35555 | Fibrillin-1 | -2.694952 |
| 35 | Q07507 | Dermatopontin | -2.576035 |
| 36 | Q92743 | Serine protease HTRA1 | -2.551748 |
| 37 | Q14203 | Dynactin subunit 1 | -2.546714 |
| 38 | Q02252 | Methylmalonate-semialdehyde dehydrogenase [acylating], mitochondrial | -2.540426 |

PXG, pseudoexfoliation glaucoma
