# Supplementary material for: Global and Comparative Proteome Signatures in the Lens Capsule, Trabecular Meshwork, and Iris of Patients With Pseudoexfoliation Glaucoma
Source: Front Mol Biosci. 2022 Apr 20;9:877250. doi: 10.3389/fmolb.2022.877250 (PMC9065473; doi:10.3389/fmolb.2022.877250)
Supplement: Supplementary file 5 [file Table4.DOCX]

**Table S4.** Top over-expressed and under-expressed proteins in trabecular meshwork (TM) with PXG compared to control.

| **Over-expressed proteins > 2.5 folds** | | | |
| --- | --- | --- | --- |
| **Sl no.** | **Accession number** | **Proteins name** | **Abundance ratio Log2**  **(PXG v/s Control)** |
| 1 | Q00325 | Phosphate carrier protein, mitochondrial | 2.5194607 |
| 2 | P26599 | Polypyrimidine tract-binding protein 1 | 2.5280629 |
| 3 | P01834 | Ig kappa chain C region | 2.5636295 |
| 4 | Q00839 | Heterogeneous nuclear ribonucleoprotein U | 2.6316713 |
| 5 | P36955 | Pigment epithelium-derived factor | 2.6417981 |
| 6 | P24821 | Tenascin | 2.6488527 |
| 7 | P02763 | Alpha-1-acid glycoprotein 1 | 2.6542736 |
| 8 | P01860 | Ig gamma-3 chain C region | 2.6725858 |
| 9 | P52895 | Aldo-keto reductase family 1 member C2 | 2.6853985 |
| 10 | P30041 | Peroxiredoxin-6 | 2.7284234 |
| 11 | P37802 | Transgelin-2 | 2.7440669 |
| 12 | P12956 | X-ray repair cross-complementing protein 6 | 2.8641374 |
| 13 | Q9UQP3 | Tenascin-N | 2.873601 |
| 14 | P14174 | Macrophage migration inhibitory factor | 2.879434 |
| 15 | P00918 | Carbonic anhydrase 2 | 2.9023355 |
| 16 | Q6UXI7 | Vitrin | 2.9133698 |
| 17 | P00558 | Phosphoglycerate kinase 1 | 2.9236398 |
| 18 | P29401 | Transketolase | 2.9544676 |
| 19 | P62258 | 14-3-3 protein epsilon | 2.9749611 |
| 20 | P27797 | Calreticulin | 2.9949002 |
| 21 | P01042 | Kininogen-1 | 2.996732 |
| 22 | P13671 | Complement component C6 | 3.0028266 |
| 23 | O43827 | Angiopoietin-related protein 7 | 3.0154057 |
| 24 | P06733 | Alpha-enolase | 3.0188107 |
| 25 | Q9UBM4 | Opticin | 3.0450802 |
| 26 | P04217 | Alpha-1B-glycoprotein | 3.1150747 |
| 27 | P25311 | Zinc-alpha-2-glycoprotein | 3.1559602 |
| 28 | P62736 | Actin, aortic smooth muscle | 3.183164 |
| 29 | P07358 | Complement component C8 beta chain | 3.1849988 |
| 30 | P07237 | Protein disulfide-isomerase | 3.2074819 |
| 31 | P30043 | Flavin reductase (NADPH) | 3.2121644 |
| 32 | P00746 | Complement factor D | 3.2403448 |
| 33 | P30838 | Aldehyde dehydrogenase, dimeric NADP-preferring | 3.2546387 |
| 34 | P30101 | Protein disulfide-isomerase A3 | 3.2660832 |
| 35 | P40925 | Malate dehydrogenase, cytoplasmic | 3.307984 |
| 36 | P62937 | Peptidyl-prolyl cis-trans isomerase A | 3.3427149 |
| 37 | P30086 | Phosphatidylethanolamine-binding protein 1 | 3.3519961 |
| 38 | Q04828 | Aldo-keto reductase family 1 member C1 | 3.3596991 |
| 39 | O75874 | Isocitrate dehydrogenase [NADP] cytoplasmic | 3.4313935 |
| 40 | P02768 | Serum albumin | 3.4848846 |
| 41 | Q00341 | Vigilin | 3.6600154 |
| 42 | P60174 | Triosephosphate isomerase | 3.7819989 |
| 43 | P06310 | Ig kappa chain V-II region RPMI 6410 | 3.8170854 |
| 44 | P07315 | Gamma-crystallin C | 3.8372069 |
| 45 | P02489 | Alpha-crystallin A chain | 3.9445764 |
| 46 | P07858 | Cathepsin B | 3.9498809 |
| 47 | P02730 | Band 3 anion transport protein | 4.0494702 |
| 48 | P49773 | Histidine triad nucleotide-binding protein 1 | 4.0685591 |
| 49 | P62826 | GTP-binding nuclear protein Ran | 4.0760595 |
| 50 | P02790 | Hemopexin | 4.1800844 |
| 51 | Q7Z7G0 | Target of Nesh-SH3 | 4.1859539 |
| 52 | P19652 | Alpha-1-acid glycoprotein 2 | 4.311735 |
| 53 | P02787 | Serotransferrin | 4.3282757 |
| 54 | P00738 | Haptoglobin | 4.3559005 |
| 55 | P02675 | Fibrinogen beta chain | 4.6706776 |
| 56 | P02656 | Apolipoprotein C-III | 4.9156657 |
| 57 | P07195 | L-lactate dehydrogenase B chain | 4.9482192 |
| 58 | P02671 | Fibrinogen alpha chain | 5.2362396 |
| 59 | P43320 | Beta-crystallin B2 | 5.6464338 |
| 60 | P05813 | Beta-crystallin A3 | 5.7913665 |
| 61 | P16402 | Histone H1.3 | 5.901063 |
| 62 | P68871 | Hemoglobin subunit beta | 5.9435444 |
| 63 | P07320 | Gamma-crystallin D | 6.9531654 |
| 64 | P22914 | Beta-crystallin S | 7.2490775 |
| 65 | P69905 | Hemoglobin subunit alpha | 7.2819464 |
| 66 | P05204 | Non-histone chromosomal protein HMG-17 | 10.269924 |
| **Under-expressed proteins <-2.5 folds** | | | |
| 1 | P24844 | Myosin regulatory light polypeptide 9 | -6.3668109 |
| 2 | Q14515 | SPARC-like protein 1 | -6.2034806 |
| 3 | P02538 | Keratin, type II cytoskeletal 6A | -5.4241718 |
| 4 | P49755 | Transmembrane emp24 domain-containing protein 10 | -5.2431002 |
| 5 | P58215 | Lysyl oxidase homolog 3 | -5.0685072 |
| 6 | Q03692 | Collagen alpha-1(X) chain | -4.9429801 |
| 7 | Q02218 | 2-oxoglutarate dehydrogenase, mitochondrial | -4.7760442 |
| 8 | P16615 | Sarcoplasmic/endoplasmic reticulum calcium ATPase 2 | -4.6971382 |
| 9 | P50895 | Basal cell adhesion molecule | -4.5147136 |
| 10 | Q9NZM1 | Myoferlin | -4.4855222 |
| 11 | P13073 | Cytochrome c oxidase subunit 4 isoform 1, mitochondrial | -4.3479034 |
| 12 | O00217 | NADH dehydrogenase [ubiquinone] iron-sulfur protein 8, mitochondrial | -4.2785017 |
| 13 | Q02543 | 60S ribosomal protein L18a | -4.2270908 |
| 14 | Q13753 | Laminin subunit gamma-2 | -4.2268923 |
| 15 | P49411 | Elongation factor Tu, mitochondrial | -4.1263606 |
| 16 | Q5JWF2 | Guanine nucleotide-binding protein G(s) subunit alpha isoforms XLas | -4.1108727 |
| 17 | Q99623 | Prohibitin-2 | -3.9432122 |
| 18 | P02533 | Keratin, type I cytoskeletal 14 | -3.9297587 |
| 19 | P08559 | Pyruvate dehydrogenase E1 component subunit alpha, somatic form, mitochondrial | -3.9234559 |
| 20 | P20674 | Cytochrome c oxidase subunit 5A, mitochondrial | -3.8888439 |
| 21 | P11177 | Pyruvate dehydrogenase E1 component subunit beta, mitochondrial | -3.8461919 |
| 22 | P05556 | Integrin beta-1 | -3.8081574 |
| 23 | P35625 | Metalloproteinase inhibitor 3 | -3.7150972 |
| 24 | P63000 | Ras-related C3 botulinum toxin substrate 1 | -3.6411484 |
| 25 | P30837 | Aldehyde dehydrogenase X, mitochondrial | -3.5771165 |
| 26 | Q6PCB0 | von Willebrand factor A domain-containing protein 1 | -3.5546956 |
| 27 | P04843 | Dolichyl-diphosphooligosaccharide--protein glycosyltransferase subunit 1 | -3.5440578 |
| 28 | P10515 | Dihydrolipoyllysine-residue acetyltransferase component of pyruvate dehydrogenase complex, mitochondrial | -3.4854664 |
| 29 | Q02978 | Mitochondrial 2-oxoglutarate/malate carrier protein | -3.4327142 |
| 30 | P25067 | Collagen alpha-2(VIII) chain | -3.4230392 |
| 32 | P39900 | Macrophage metalloelastase | -3.4101174 |
| 33 | O60814 | Histone H2B type 1-K | -3.3550143 |
| 34 | P21796 | Voltage-dependent anion-selective channel protein 1 | -3.3487058 |
| 35 | P56134 | ATP synthase subunit f, mitochondrial | -3.3401687 |
| 36 | Q9HBL0 | Tensin-1 | -3.3341566 |
| 37 | O95168 | NADH dehydrogenase [ubiquinone] 1 beta subcomplex subunit 4 | -3.3274097 |
| 38 | P05091 | Aldehyde dehydrogenase, mitochondrial | -3.288258 |
| 39 | Q14315 | Filamin-C | -3.2646025 |
| 40 | P24539 | ATP synthase F(0) complex subunit B1, mitochondrial | -3.2416612 |
| 41 | Q07065 | Cytoskeleton-associated protein 4 | -3.2275882 |
| 42 | Q13683 | Integrin alpha-7 | -3.1829335 |
| 43 | P04899 | Guanine nucleotide-binding protein G(i) subunit alpha-2 | -3.1772248 |
| 44 | P31930 | Cytochrome b-c1 complex subunit 1, mitochondrial | -3.1426886 |
| 45 | O43181 | NADH dehydrogenase [ubiquinone] iron-sulfur protein 4, mitochondrial | -3.1140457 |
| 46 | P21912 | Succinate dehydrogenase [ubiquinone] iron-sulfur subunit, mitochondrial | -3.1111315 |
| 47 | Q13885 | Tubulin beta-2A chain | -3.0708265 |
| 48 | P10606 | Cytochrome c oxidase subunit 5B, mitochondrial | -3.0665773 |
| 49 | Q9NQ79 | Cartilage acidic protein 1 | -3.0629527 |
| 50 | P29034 | Protein S100-A2 | -3.05677 |
| 51 | P22352 | Glutathione peroxidase 3 | -3.0071311 |
| 52 | P60033 | CD81 antigen | -2.9831323 |
| 53 | Q07092 | Collagen alpha-1(XVI) chain | -2.9598941 |
| 54 | Q9Y3Z3 | Deoxynucleoside triphosphate triphosphohydrolase SAMHD1 | -2.9447761 |
| 55 | P13647 | Keratin, type II cytoskeletal 5 | -2.9408246 |
| 56 | P12236 | ADP/ATP translocase 3 | -2.9324629 |
| 57 | P35908 | Keratin, type II cytoskeletal 2 epidermal | -2.9280603 |
| 58 | P00387 | NADH-cytochrome b5 reductase 3 | -2.9070465 |
| 59 | P48047 | ATP synthase subunit O, mitochondrial | -2.8688179 |
| 60 | P35580 | Myosin-10 | -2.8072575 |
| 61 | Q16891 | MICOS complex subunit MIC60 | -2.6856996 |
| 62 | P55001 | Microfibrillar-associated protein 2 | -2.6813301 |
| 63 | P31151 | Protein S100-A7 | -2.6549507 |
| 64 | P30048 | Thioredoxin-dependent peroxide reductase, mitochondrial | -2.6016231 |
| 65 | Q13418 | Integrin-linked protein kinase | -2.5945799 |
| 66 | P08311 | Cathepsin G | -2.5900674 |
| 67 | Q562R1 | Beta-actin-like protein 2 | -2.5815718 |
| 68 | P09471 | Guanine nucleotide-binding protein G(o) subunit alpha | -2.5720109 |
| 69 | Q13214 | Semaphorin-3B | -2.5642283 |
| 70 | P81605 | Dermcidin | -2.5379385 |
| 71 | O43920 | NADH dehydrogenase [ubiquinone] iron-sulfur protein 5 | -2.5079463 |

PXG, pseudoexfoliation glaucoma
